# Numerical Analysis and Poromechanics Calculation for Saturated Mortar Involved with Sub-Freezing Temperature

**DOI:** 10.3390/ma15227885

**Published:** 2022-11-08

**Authors:** Wei Xie, Huaizhi Su, Chenfei Shao, Sen Zheng

**Affiliations:** 1 State Key Laboratory of Hydrology-Water Resources and Hydraulic Engineering, Hohai University, Nanjing 210098, China; 2 College of Water Conservancy and Hydropower Engineering, Hohai University, Nanjing 210098, China; 3 College of Civil and Transportation Engineering, Hohai University, Nanjing 210098, China

**Keywords:** saturated mortar, freeze-thaw process, pore pressure, temperature distribution, numerical analysis

## Abstract

The individual coupling processes of two-phase materials are controlled to some extent by damage theory. However, the existing theory is not sufficient to explain the effect of pore pressure on mortar materials under freeze-thaw action. In order to predict the resistance of saturated mortars during rapid cooling and to describe the physical behavior of the pore structure, the authors derived in detail the governing equations of saturated mortars during freezing in the framework of the pore elasticity theory and analyzed the sensitivity of physical parameters to the influence of temperature stresses by means of stress-strain calculations. In addition, the effects of phase change and latent heat of freezing on the local thermodynamic equilibrium are considered, and a mathematical model is established for quantitatively simulating the temperature distribution of the specimen. This model is reformulated and extended in the current work to intuitively reveal the effect of concrete dimensions on the temperature hysteresis effect. The results of the numerical model calculations show that during the freezing process, for the specimen with dimensions of 50 mm × 50 mm × 50 mm and a water-cement ratio of 0.6, the maximum temperature difference from center to surface is 10 °C, the maximum vertical strain on the surface is 4.27 × 10^−4^, and the maximum pore water pressure at the center of the specimen is 76 MPa. The model calculation results present a similar pattern to the physical interpretation and reference results, thus effectively evaluating the freezing damage process of saturated mortar.

## 1. Introduction

As a common porous building material, engineering concrete is widely subjected to complex and uncertain conditions including large temperature variations and high frequency periodicity in cold regions, which make frost damage a very common difficulty in dams, locks and other hydraulic concrete structures. Over the past century, the research on the performance degradation caused by the freeze-thaw process on concrete has made great progress [1]. It has been proved that the frost damage caused by pressure involves the transfer of inside liquid water, which is attributed to the expansion process of ice crystals. Similar attributions also include hydrostatic pressure, osmotic pressure, fatigue stress, bond spalling, etc. According to these above attributions, the main reasons for mortar performance deterioration can be classified into boundary conditions, thermodynamic parameters, water saturation, characteristics and distribution of pore structure [2,3,4]. However, the existing theories show that it is still difficult to avoid all frost damage in concrete [5,6]. The stress caused by the temperature cycle and humidity fluctuation will lead to cracks inside the concrete structure. The integrity, stability and durability of the system will decrease with the loss of strength and stiffness, and the stress can even induce foundation penetration cracks in the structure [7].

As a heterogeneous material, cement paste is composed of irregular admixtures, various hydration products and residual pores [8,9]. The size distribution of concrete pores may span seven orders of magnitude (1~10^7^ nm) [10]. The heterogeneity is enhanced by the presence of aggregate, which is significantly different from the pore structure of the surrounding hydrated cement paste, and affects the pore distribution in the interface transition zone. In order to obtain the pore characteristics and thermodynamic properties of materials, the systematic experimental techniques, such as nuclear magnetic resonance (NMR), scanning electron microscope (SEM), computerized tomography (CT), thermal-mechanical analysis, ultrasonic and dielectric measurements are applied to form many frost damage theories, which is widely used to elaborate the damage mechanism of saturated mortar to explain all phenomena with sub-freezing [11,12,13]. The CT technology adopted in this paper can effectively realize the dual identification of pore structure and the moisture condition. Both local macro damage and micro damage can be deduced by analyzing the characteristics and evolution law of microstructure damage during the frost process. At the same time, CT technology can provide a three-dimensional reconstruction model for numerical simulation [14].

Numerical analysis is a more suitable approach for the calculation of three-dimensional structures under the action of coupling fields in complex working conditions [15]. Olsen [16] designed a two-dimensional finite element model considering the influence of humidity, temperature, pore pressure and other factors to simulate the freeze-thaw process of concrete under the condition of water saturation. With the stress-strain relationship of multiple pore systems as a foundation, Bazant [17] conducted tests and measured the relative humidity and temperature at the center and surface of specimens. Although the relationship between pore pressure and strain and equivalent stress was introduced to establish a mathematical model for predicting the frost durability of concrete, the model has not been widely adopted in practical engineering due to the difficulty in calibrating parameters of the model and the great difference from the actual measured values to the model results. In addition, the thermodynamics viewpoint is introduced into the elastic pore theory, and the expression of the relation between pore pressure, stress strain and temperature is put forward by Coussy [18].

From the view of the micro level, the simulation of the freeze-thaw process of fully saturated mortar has been extensively discussed in the two-dimensional model by Zuber [19]. On the basis above, we expanded a set of 2D control equations of freezing process to a 3D model, which consists of differential equations involving matrix deformation, the transform pressure between water and ice formation along with the temperature field distribution in pores. These equations are applied for the numerical rule of the volume strain distribution in porous materials with the freezing temperature field [20]. The complexity of the equations lies in the coupling relationship between the liquid transfer and ice formation within the pore structure, and the pore structure stress response exposed to the temperature boundary during the cooling process.

Practical experience shows that the finite element method can integrate the temperature, structure and seepage fields into a unified program for calculation, and can adapt to complex irregular boundary conditions. Nevertheless, the THM coupling problem during frost has received less attention so far. The coupling effect can be decomposed into the following three main contents: the seepage control equation can be used to study the water immersion path and water storage state; the thermal conduction equation tends to simulate the physical effect of heat on pore water suffered with the rapid drop of temperature; the stress-strain relationship in the two-phase pore structure was solved by a physical mechanics equation. In this work, a new three-field coupled model, which was used as an extension and recalculation method of the two-dimensional model, was proposed to obtain a more reasonable simulation of the physical phenomena during frost [21].

In the first part of this paper, the relationship between the freezing point of pore water and the critical radius of frozen pores is briefly reviewed and extended by deducing the loads borne on the pore structure of porous mortar under freeze-thaw conditions, so as to accurately reproduce the influence of the freezing process on the mechanical properties of material. Then, by using extensive derivations in the literature, from general physical formulae through to parameter’s definition, a coupled THM model for calculating pore water pressure under freezing conditions is proposed. In particular, the local thermodynamic equilibrium equation, which takes into account the effect of the latent heat of phase transition on the temperature field is considered to show the applicability of the model in reproducing the main effects of freezing damage on concrete properties. Finally, a 3D model of a 50 mm × 50 mm × 50 mm concrete member was reconstructed with CT technology, and the finite element numerical simulation was carried out to verify the ability of the model to capture the experimental behavior of the member.

By studying the existing damage mechanism of freeze-thaw and the feedback of the pore structure to the thermodynamic boundary, the mix proportion design of concrete composition under different working conditions can be optimized through exploring the huge difference of water transport under different pore structures, which is conducive to finding feasible measures to reduce freezing damage, such as the research and development of air entrained concrete [22,23]. The establishment of reasonable numerical simulation of freeze-thaw damage mortar needs to be considered comprehensively from the micro-mechanics to overall behavior, which is still in the exploration stage for more attention.

## 2. Characteristics and Mechanics of Porous Mortar

### 2.1. Freezing Point with the Critical Radius in Spherical Pores

The pore distribution in mortar specimens can be obtained according to the mercury injection method (MIP) [8], and the pores can be divided into four major categories, respectively gel pores in cement material, capillary pores, entrained air void, and entrapped air at millimeter-level. According to the thermodynamic theory derived by Fagerlund and Hinman [24,25], compared with the total pores in fully saturated concrete, the freezing volume change of moderately saturated mortar specimens is negligible, due to the existing provides sufficient space to sustain the 9% expansion of crystallization [26,27]. At the same time, due to the service environment of hydraulic concrete, especially the area with water level fluctuation, this paper assumes that the porous materials always maintain a water saturation state. The equilibrium expression for freezing point depression during the formation of spherical ice crystals can be expressed as follows [24]:

(1) $$\ln\frac{T_{0}-\Delta T}{T_{0}}=-\omega \cdot \frac{2\cdot \sigma_{sl}\cdot M}{\rho_{l}\cdot \Delta H}\cdot \frac{1}{r_{sl}}+\left(\frac{1}{\rho_{l}}-\frac{1}{\rho_{s}}\right)\cdot \frac{2\cdot \sigma_{sg}\cdot M}{\Delta H}\cdot \frac{1}{r_{sg}}$$

where $T_{0}$ is the normal freezing point, 273.15 K, ΔT is the decrement of freezing point with the change of radius, M is the molar mass with the unit kg, ω is the form factor of different ice crystal, and the value ranges in ω∈(1.1,1.3), $r_{sl}$ is the freezing equilibrium radius with the unit m, ΔH is the molar heat of phase transition in water with the unit kJ, $\rho_{l}$ is the density of water decreases with temperature with the unit kg/m^3^, and $\sigma_{sl}$ is the water-ice interfacial tension, the surface tension factor of which could be written as about 36 × 10^−3^ N/m [19].

The water in the closed pore can be regarded as a single component system without considering the other trace substances contained within it. Due to the restraint of the closed pore wall, the expansion from transformation is confined, which leads to partial subcooled water in pores. At this point, the coexistence of water and ice in the pore follow the relationship between the phase number, temperature and pressure into an equilibrium.

The medium is assumed to remain saturated completely without the vapor phase filled in, that is to say, the extra pressure in the solid/gas interface $\sigma_{sg}$ is limited to zero, affected with the positive crystal radius, $r_{sg}\to \infty$, which demonstrates that for the second term, the influence of saturated vapor pressure could be negligible. The uniform compressive stress acting on the inner surface of the closed pore can be obtained [24].

(2) $$\Delta p_{sl}=\frac{2\cdot \sigma_{sl}}{r_{sl}}=-\frac{\rho_{l}\cdot \Delta H}{\omega \cdot M}\cdot \ln\frac{T_{0}-\Delta T}{T_{0}}\approx \frac{\rho_{s}\cdot \Delta H\cdot \Delta T}{\omega \cdot M\cdot T_{0}}$$

Compare it with the Clapeyron equation, where ΔH is the molar heat of phase transition, 333.5 KJ, with spherical crystal ω=1 [17], the approximate expression is valid at temperatures around 0 °C. Then the critical radius $r_{sl}$ in water/solid interface was derived as follows:

(3) $$r_{sl}=-\frac{2\cdot \sigma_{sl}}{\rho_{l}\cdot \Delta H}\cdot \frac{M}{\ln\frac{T_{0}-\Delta T}{T_{0}}}=-\frac{2\cdot \sigma_{sl}}{\rho_{l}\cdot H_{l}\cdot \ln\frac{T_{0}-\Delta T}{T_{0}}}\approx \frac{2\cdot \sigma_{sl}}{\rho_{s}\cdot H_{l}}\cdot \frac{T_{0}}{\Delta T}$$

where $H_{l}=\frac{\Delta H}{M}$ is defined by the latent heat and the molar mass, usually as the heat flux during phase transition with the unit kJ/kg, ΔT is the drop of freezing point with unit K.

With the decrease of temperature and the influence of saturated vapor pressure, the freezing point decreases with the decrease of pore size, and ice crystal gradually forms in the larger pores firstly and further penetrates into the smaller pores in the structure. Above the capillary pore size, the solidification of the pressure water has a characteristic called super-cooling, which appears as the lower the freezing point, the greater the pressure, probably in −3 °C~−4 °C. The cementing pore size is so small with hardly a freezing point below −80 °C which suggests that the liquid has a non-freezing nature as adsorption water [28,29].

Cement paste is a material with very small pores, in the case of non-limit low temperature, the pore wall of the material, i.e., the surface of the ice crystal, retains a layer of unfrozen adsorbed water film which induces to decrease the effective radius, and the thickness of water film is a function of temperature. At 0 °C, the thickness of the water film on the clay particles is 70 Å, Å=0.1 nm is a constant diameter theoretically as one quarter of the water molecule, and the value of the glass material is 50 Å [30].

In order to accurately evaluate pore size distribution, Powers deduced the connection between temperature and the thickness of the adsorbed layer based on Powers’ concrete adsorption theory [31], the thickness of adsorption layer was modified into $\delta =1.97{\left|\Delta T\right|}^{-\frac{1}{3}}$, 1.97 is a constant with the unit $K^{1/3}\cdot \mathrm{nm}$. Thus, when the loading temperature reaches ΔT, the pores in the structure with a radius larger than $R_{eq}$ will be frozen while smaller water pores stay in the liquid.

(4) $$R_{eq}=r_{sl}+\delta =\frac{2\cdot \sigma_{sl}\cdot T_{0}}{\Delta \rho \cdot H_{l}\cdot \Delta T}+1.97\cdot \sqrt[{3}]{\frac{1}{\left|\Delta T\right|}}$$

According to the measurement results, At ΔT=0.05 °C, δ=53 Å, and ΔT=0.2 °C, δ=34 Å are in accordance with the values stated above.

### 2.2. Theoretical Calculation of Water Pressure in Spherical Pores

Spherically symmetric problems can be defined as those in which the geometry, constraints and external factors of an elastic body are all symmetric to the same point, resulting in all stresses, deformations and displacements being symmetric to this point. According to the characteristics of spherical symmetry, the spherical coordinates should be used with r. If the symmetric point of the elastic body is taken as the origin of coordinates, the stress component, deformation component and displacement component of the spherical symmetry problem are only functions of the radial coordinates and have nothing to do with the other two coordinates. Obviously, spherical symmetry problems can only occur in hollow or solid round spheres. If the symmetric point of the elastic body is taken as the origin of coordinates, the stress component, deformation component and displacement component of the spherical symmetry problem are only functions of the radial coordinates and have nothing to do with the other two coordinates. Obviously, spherical symmetry problems can only occur in hollow or solid round spheres [32,33,34].

The stress calculation of spherical pore in this paper is based on three assumptions. The hollow spheres pores are surrounded by a certain thickness of cement mortar, as an isotropy elasticity material, mortar is continuous and uniform. The inner surface of the ball is subjected to the ice pressure uniformly.

**Figure 1:**
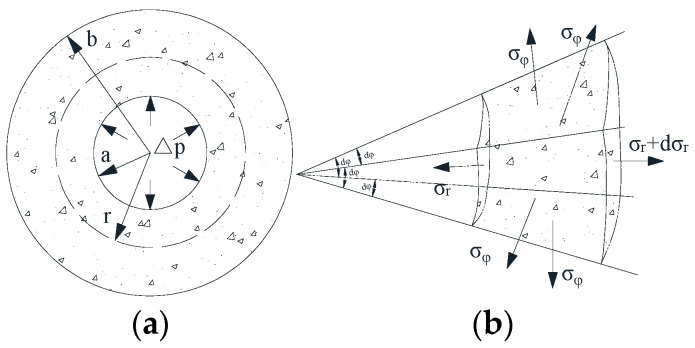
(**a**) Internal surface force diagram of pores; (**b**) Force balance diagram of micro element.

According to the spherically symmetric problem in elastic mechanics, a micro hexahedron was cut from the elastomer using two pairs of radial planes with intersecting angles dφ each other, and two separate spheres dr apart with only normal stress on each surface, as shown in Figure 1.

On the basis of the radial balance $F_{r}+F_{T}=F_{r+dr}$, the equilibrium differential equation of the spherical symmetry problem can be obtained as follows with the sinusoidal variation $\sin\frac{d\varphi }{2}=\frac{d\varphi }{2}$ when the value dφ is small enough, while the second order micro amounts are omitted [35]. The equilibrium differential equations of spherically symmetric problems can be obtained as below:

(5) $$\frac{d\sigma_{r}}{dr}+\frac{2}{r}\left(\sigma_{r}-\sigma_{\varphi }\right)+F_{r}=0$$

In addition, for the same reason, the symmetry of spherical pores leads to the simple balance with only radial displacement, radial and tangential positive strain, but the shear strain at the coordinate direction has no chance of occurring. By combining the geometric equation and physical equation of the spherically symmetric problem derived directly from Hooke’s law, the elastic equation can be expressed as:

(6) $$\{\begin{array}{l} \varepsilon_{r}=\frac{du_{r}}{dr}=\frac{1}{E}\left(\sigma_{r}-2\mu \sigma_{\varphi }\right)\\ \varepsilon_{\varphi }=\frac{u_{r}}{dr}=\frac{1}{E}\left[\left(1-\mu \right)\sigma_{\varphi }-\mu \sigma_{r}\right] \end{array}\Rightarrow \{\begin{array}{l} \sigma_{r}=\frac{E}{\left(1+\mu \right)\left(1-2\mu \right)}\left[\left(1-\mu \right)\frac{du_{r}}{dr}+2\mu \frac{u_{r}}{dr}\right]\\ \sigma_{\varphi }=\frac{E}{\left(1+\mu \right)\left(1-2\mu \right)}\left(\frac{u_{r}}{dr}+\mu \frac{du_{r}}{dr}\right) \end{array}$$

Owing to the absence of the only radial volume force $K_{r}=0$, based on the displacement $u_{r}$, the basic differential equation applied to solve the spherically symmetric problem in terms of displacements can be obtained through substituting Equation (6) into Equation (5). With a hollow sphere, the inner radius is *a*, the outer radius is *b*, the internal pressure is $q_{a}$, the external pressure is $q_{b}$, then its stress and displacement can be obtained:

(7) $$\frac{d^{2}u_{r}}{dr^{2}}+\frac{2}{r}\frac{du_{r}}{dr}-\frac{2}{r^{2}}\mu_{r}=0\;\Rightarrow \;\{\begin{array}{c} u_{r}=Ar+\frac{B}{r^{2}}\\ \sigma_{r}=\frac{E}{1-2\mu }A-\frac{2E}{1+\mu }\frac{B}{r^{3}}\\ \sigma_{\varphi }=\frac{E}{1-2\mu }A+\frac{2E}{1+\mu }\frac{B}{r^{3}} \end{array}$$

An initial boundary condition of temperature is T=10 °C (283.15 K) with the normal freezing temperature $T_{1}=0$ °C (273.15 K) and the freezing temperature with pore pressure taken as $\Delta T_{1}=-2$ °C (271.15 K), both of which are adopted for calculations, then the compressive stress, which is evenly distributed on the inner wall can be described on the basis of the Clapeyron equation [36] as ${\left(\sigma_{r}\right)}_{r=a}=q_{a}=\frac{\Delta \rho \cdot \Delta H\cdot \Delta T}{\omega \cdot M\cdot T_{0}}=-27.07\;\mathrm{MPa}$ at the radius r=a, and ${\left(\sigma_{r}\right)}_{r=b}=q_{b}=0$ at the radius r=b. Substituting into the above solution. Knowing that

(8) $$A=\frac{a^{3}q_{a}-b^{3}q_{b}}{E\left(b^{3}-a^{3}\right)}\left(1-2\mu \right)=\frac{a^{3}\left(1-2\mu \right)}{E\left(b^{3}-a^{3}\right)}\cdot q_{a},B=\frac{a^{3}b^{3}\left(q_{a}-q_{b}\right)}{2E\left(b^{3}-a^{3}\right)}\left(1+\mu \right)=\frac{a^{3}b^{3}\left(1+\mu \right)}{2E\left(b^{3}-a^{3}\right)}q_{a}$$

Thus, the expressions of radial displacement and stress in the spherically symmetric problem were derived as follows.

(9) $$\{\begin{array}{c} \sigma_{r}=\frac{\left(b^{3}-r^{3}\right)a^{3}}{r^{3}\left(b^{3}-a^{3}\right)}\cdot q_{a}\\ \sigma_{\varphi }=\frac{\left(a^{3}b^{3}+2a^{3}r^{3}\right)}{2r^{3}\left(b^{3}-a^{3}\right)}\cdot q_{a}\\ u_{r}=\left(\frac{2r^{3}a^{3}\left(1-2\mu \right)+a^{3}b^{3}\left(1+\mu \right)}{2r^{2}E\left(b^{3}-a^{3}\right)}\right)\cdot q_{a} \end{array}\;$$

The specific surface area and air content are $\alpha =31.4mm^{-1},C=3.61\%$ respectively. The number of spherical pores in cement stone $N=\frac{C}{\frac{4}{3}\pi a^{3}}=\frac{3.61\%}{\frac{4}{3}\pi {\left(0.096\times 10^{-3}\right)}^{3}}=9.88\times 10^{9}$ with the pore radius a=3/α=0.096 mm. It is assumed that the spherical pores are evenly distributed in the cement stones, and the volume of the cement stones is taken as p=29.2%, then the spherical outer radius is half the diagonal of the cube which contains a pore as $b=\frac{\sqrt{3}}{2}\sqrt[{3}]{\frac{p+C}{N}}=0.278mm$, then the pore spacing factor gets L=b−a=0.183mm as the maximum distance from any point in the cement to any adjacent bubble sphere. The tensile stress of the inner pore wall is similarly obtained as the tensile stress of the outer wall.

(10) $$\sigma_{\varphi }^{a}=\frac{\left(a^{3}b^{3}+2a^{3}a^{3}\right)}{2a^{3}\left(b^{3}-a^{3}\right)}\cdot q_{a}=-15.25\;\mathrm{MPa},\;\sigma_{\varphi }^{b}=\frac{\left(a^{3}b^{3}+2a^{3}b^{3}\right)}{2b^{3}\left(b^{3}-a^{3}\right)}\cdot q_{a}=-1.71\;\mathrm{MPa}$$

where *a* could be taken as a control of the pore radius, *b* is on behalf of the hole spacing, and $q_{a}$ represents the influence of freezing temperature. Based on which, the sensitivity analysis of the effect factors can be carried out on the pore stress of cement mortar. It is worth emphasizing that the effect of pore spacing on the tensile stress is strongly related to the openness of pores. In the connected pore, however, the stress will increase with the ascending space, which is totally different from the phenomenon in the closed pore, probably because the spacing will hinder the water migration when the water freezes and generates pressure, thus increasing the tensile stress.

## 3. Mass Conservation and Phase Equilibria during Ice Formation

The critical phase transition temperature of pore water scales down with the decrease of pore size. In the freezing process in fully saturated pores, according to mass conservation, the variable quantity of liquid water is the sum of consumption due to the Darcy-flow and freezing. Assuming that the specimen is a water storage model with no internal flow, the increase mass of ice is equal to the consume mass of liquid [37], and $V_{s}+V_{l}=1$, then the relation between the consume volume of liquid and the increase volume of ice can be written as:

(11) $$\frac{dV_{l}}{dt}=-\frac{dV_{s}}{dt}\Rightarrow \frac{n}{V_{l}}\frac{dV_{l}}{dt}=-\frac{V_{s}}{V_{l}}\frac{n}{V_{s}}\frac{dV_{s}}{dt}$$

where n is the porosity, dt is the time increment, $V_{l},V_{s}$ is the volumetric fraction of liquid and crystal, respectively.

Assuming that the concrete remains fully saturated with water in ideal spheres, and ice fills the remaining pores at any given freezing temperature, the pore volume content of the adsorption layer is $\frac{4\pi R^{2}\cdot \delta }{3/4\pi R^{3}}=\frac{3\delta }{r}$, and the total volume of the pore adsorption layer above the critical radius can be expressed as $V_{a}=\int_{R_{eq}}^{\infty }\frac{3\delta }{r}\frac{dO}{dr}dr$, where $V_{s}+V_{l}=1$, and $V_{s}$, $V_{l}$ are respectively the proportions of ice and water in pores, n is the total porosity, $V_{l\to s}$ is the variable volume from water to ice in the pores when the freezing point is ΔT, notice that $V_{l\to s}$ is also the difference between the accumulated volume of frozen pores and the volume of the adsorption layer, and can be expressed as:

(12) $$V_{l\to s}=O\left(R_{eq}\right)-V_{a}=O\left(R_{eq}\right)-\int_{R_{eq}}^{\infty }\frac{3\delta }{r}\frac{dO}{dr}dr$$

In the formula, the pore data of the mortar specimen with a water-cement ratio of 0.6 was collected by the mercury injection method (MIP), and calculated and analyzed for the fitting curve of the pore diameter distribution which was obtained as $\frac{dO}{dr}=9.366\times 10^{-4}+\frac{4.9389\times 10^{-6}}{1+{\left(R_{eq}/95.80985\right)}^{2.27832}}$ [38]. As the temperature reaches below freezing point, the freezing amount for each 1 °C reduction, freezing rate ${\dot{\omega }}_{l\to s}$ leads into a rapid growth stage.

(13) $${\dot{\omega }}_{l\to s}=\frac{dm_{s}}{dT}=\rho_{l}\frac{dV_{l\to s}}{dT}=\rho_{l}\frac{dV_{l\to s}}{dr}\frac{dr}{dT}=\rho_{l}\left(\frac{dO}{dr}-\frac{dV_{a}}{dr}\right)\frac{dr}{dT}=\rho_{l}\left(1-\frac{3\delta }{r}\right)\frac{dO}{dr}\frac{dr}{dT}$$

where $m_{s}$ is the freezing mass of water, and $\rho_{l}$ is the density of water.

The consume volume of liquid can be obtained as follows:

(14) $$\frac{n}{K_{l}}{\dot{p}}_{l}+\frac{b_{i}-n}{K_{m}}{\dot{p}}^{*}-[n\alpha_{l}+((b_{i}-n)\alpha_{o})]\dot{T}+b_{i}\dot{\varepsilon }+\frac{n}{V_{l}}\frac{dV_{l}}{dt}+\frac{1}{V_{l}}div(j_{l})=\frac{-{\dot{\omega }}_{l\to s}}{V_{l}\rho_{l}}$$

The increase volume of ice can be obtained as follows:

(15) $$\frac{n}{K_{s}}{\dot{p}}_{s}+\frac{b_{i}-n}{K_{m}}{\dot{p}}^{*}-[n\alpha_{s}+((b_{i}-n)\alpha_{o})]\dot{T}+\frac{n}{V_{s}}\frac{dV_{s}}{dt}+b_{i}\dot{\varepsilon }=\frac{+{\dot{\omega }}_{l\to s}}{V_{s}\rho_{s}}$$

where $K_{l},K_{s},K_{m}$ are the compressibility modulus of water, mortar and ice given by Zuber and Machand [19], ${\dot{p}}_{s},{\dot{p}}^{*}$ is the pressure from water and crystal respectively, $\alpha_{l},\alpha_{s},\alpha_{o}$ are the coefficients of linear expansion, $\dot{T}$ is the temperature decrease rate, $\dot{\varepsilon }$ is the change of the volumetric strain, $b_{i}$ is the Biot’s coefficient, usually taken as $b_{i}=1-K_{0}/K_{m}$, $K_{0}$ is the compressibility modulus of the drained porous material, ${\dot{\omega }}_{l\to s}$ is the change rate of solid mass, $j_{l}$ is the volumetric flow of liquid solution, as a fluid flux to the pressure gradient with a transfer coefficient k, $j_{l}=-k\mathrm{grad}p_{l}=-\frac{D}{\eta_{l}}\mathrm{grad}p_{l}$, D is the material permeability coefficient, $\eta_{l}$ is the dynamic viscosity of water.

According to Equation (8), substituting Equation (11) and Equation (12), it can be given as follows:

(16) $$\left(\frac{1}{\rho_{s}}-\frac{1}{\rho_{l}}\right){\dot{\omega }}_{l\to s}=\frac{nV_{l}}{K_{l}}p_{l}+\frac{nV_{s}}{K_{s}}p_{s}+\frac{b-n}{K_{m}}{\dot{p}}^{*}+b\dot{\varepsilon }-[nV_{l}\alpha_{l}+nV_{s}\alpha_{s}+(b-n)\alpha_{o}]\dot{T}+div(j_{l})$$

In order to simplify Equation (13) for the physical significance, the source term $S=\left(\frac{1}{\rho_{s}}-\frac{1}{\rho_{l}}\right){\dot{\omega }}_{l\to s}+\bar{\alpha }\dot{T}-\frac{b-n}{K_{m}}\dot{X}-\frac{nV_{s}}{K_{s}}\dot{\kappa }$, is composed of four parts. The freezing rate can be taken as ${\dot{\omega }}_{l\to s}=\rho_{l}\left(1-\frac{3\delta }{r}\right)\frac{dO}{dr}\frac{dr}{dT}$ as mentioned in Equation (10), the compound linear expansion coefficient of the system can be noted as $\bar{\alpha }=nV_{l}\alpha_{l}+nV_{s}\alpha_{s}+\left(b-n\right)\alpha_{o}$, the difference between the hydrostatic pressure and average pore pressure can be noted as $\dot{X}=p^{*}-p_{l}=\frac{\sigma_{sl}}{n}\int_{r_{sl}}^{\infty }\frac{1}{r-\delta }\frac{dO}{dr}dr$, and the pressure difference generated by capillary action from the water coupled with ice in pores can be expressed by $\dot{\kappa }=p_{s}-p_{l}=2\sigma_{sl}/r_{sl}$ [39].

According to the laws of mass conservation and Darcy’s law, water migration in the concrete system could be represented with the seepage control equation as follows [15]:

(17) $$\beta p_{l}=div(\frac{D}{\eta_{l}}\mathrm{grad}p_{l})+S-b\dot{\varepsilon }$$

where $\beta =\frac{nV_{l}}{K_{l}}+\frac{nV_{s}}{K_{s}}+\frac{b-n}{K_{m}}$ is the suggested effects result from the compressibility modulus and the pore structure distribution, $\dot{\varepsilon }$ is the change of the volumetric strain, $\alpha_{l},\alpha_{s},\alpha_{o}$ is the volume expansion coefficient of water, ice and the matrix, and $\rho_{s},\rho_{l}$ is the density of ice and water, respectively.

According to the material parameters and pore structure distribution derived in Section 2, pore water pressure is mainly affected by $\bar{\alpha }$ before freezing, and affected by freezing rate ${\dot{\omega }}_{l\to s}$ during freezing. In conclusion, the formation of ice in a pore structure triggered two effects, namely pore water pressure and structural strain during the freezing process. The pressure source of pore water with the independent temperature decrease rate $\dot{T}$.

## 4. Heat Balance upon the Sub-Freezing Process

The phase transformation and diffusion behavior of water in the mortar specimen is similar to that of a dispersed heat source or radiator. In addition to thermodynamic boundary, the rising and falling variation of the temperature field is mainly influenced by the latent heat of water during freezing-thawing in the pore space [40]. The fact that the latter two quantities contain the pore adsorption heat and heat transfer through water can be ignored in the heat conduction differential equation.

The latent heat of pore water will be released at the airside during phase transition. However, in the numerical calculation, it can be considered that the latent heat is released gradually when the transformation temperature ranges from $T_{o}-\xi$ to $T_{o}+\xi$, ξ is a small amount less than 1K near the freezing point. Therefore, the temperature control equation can generally be written as [41]:

(18) $$\rho C\frac{\partial T}{\partial t}=\lambda \mathrm{div}(\mathrm{grad}T)-\dot{H}=\lambda \cdot \nabla^{2}T+\frac{H_{l}}{2\xi }\frac{\partial \omega_{l\to s}}{\partial t}\cdot I_{T}$$

where ρ is the mortar density, $\dot{H}=333.5$ kJ/kg is the latent heat of water as phase transition, λ and C are the thermal conductivity and isobaric heat capacity of mortar, $\nabla^{2}$ is the Laplace operator, and $I_{T}=\{\begin{array}{c} 1,\\ 0, \end{array}\begin{array}{c} T_{o}-\xi <T<T_{o}+\xi \\ others \end{array}$ is a transition function.

The introduction of latent heat during phase transformation, which creates a condition for obtaining the temperature distribution of mortar specimen and the coupling relation involved water migration and strain. Because of the gradual release of latent heat, during the slow freezing-thawing process, the heat conduction process is transformed into the basic equation of solid heat conduction as $C\frac{\partial T}{\partial t}=\nabla \cdot (\lambda \nabla T)$, which is no longer within the coupling research.

According to the temperature control equation, the freezing rate, pore stress and strain at any point inside the specimen can be obtained accordingly on the basis of the instantaneous temperature distribution of the mortar specimen. In this paper, the variation method is adopted to solve the temperature field of the finite element specimen for studying the relationship between the calculated value and the experimental value.

The variation principle is a universally applicable basic conservation law in the physical world, which is also known as the principle of least action. The variation method is a mathematical programming method that takes the variation principle to access the minimum value of function under the physical constraint equation. For the space temperature field [15], the functional equation can be expressed as:

(19) $$I(T)=\underset{R}{∭}F(T,T_{x},T_{y},T_{z})dxdydz+\iint_{C}G(T)ds$$

where R is the volume fraction within the solution domain, and C is the surface integral on the boundary of the solution domain. G(T) is taken as a function of the temperature field T. $F(T,T_{x},T_{y},T_{z})$ is a function of temperature field T and temperature gradient $T_{x}=\partial T/\partial x$, $T_{y}$ and $T_{z}$. The value of the functional I(T) depends on $T,T_{x},T_{y},T_{z}$. That is to say, finding the most suitable $F(T,T_{x},T_{y},T_{z})$ and G(T) among all the temperature fields satisfies the temperature boundary conditions, so that the net heat inflow into the system is minimized.

For the spatially unstable temperature field in three-dimensional region R, the heat conduction equation can be described as:

(20) $$\frac{\partial^{2}T}{\partial x^{2}}+\frac{\partial^{2}T}{\partial y^{2}}+\frac{\partial^{2}T}{\partial z^{2}}+\frac{1}{a}\left(\frac{\partial \theta }{\partial \tau }-\frac{\partial T}{\partial \tau }\right)=0$$

where $\frac{\partial \theta }{\partial \tau }=\frac{\dot{H}}{C\rho }=\frac{WH_{l}}{C\rho }$ is the cooling rate due to the latent heat from phase transition, λ and C are the thermal conductivity and isobaric heat capacity. The temperature conductivity coefficient is a=λ/Cρ with the unit $m^{2}/h$. The initial instantaneous temperature distribution could be considered as a constant which suggests that if τ=0, $T\left(x,y,z\right)=T_{a}=constant$.

Assuming that the exothermic coefficient on the surface of the mortar specimen is large enough, the surface temperature T is equal to the air temperature $T_{a}$, which meets the first boundary condition G(T)=0. Take a function as:

(21) $$F(T,T_{x},T_{y},T_{z})=\frac{1}{2}\left[{\left(\frac{\partial T}{\partial x}\right)}^{2}+{\left(\frac{\partial T}{\partial y}\right)}^{2}+{\left(\frac{\partial T}{\partial z}\right)}^{2}\right]-\frac{1}{a}\left(\frac{\partial \theta }{\partial \tau }-\frac{\partial T}{\partial \tau }\right)=0$$

Substitute into Equation (16) and get the functional as follows:

(22) $$I\left(T\right)=\underset{R}{∭}\left\{\frac{1}{2}\left[{\left(\frac{\partial T}{\partial x}\right)}^{2}+{\left(\frac{\partial T}{\partial y}\right)}^{2}+{\left(\frac{\partial T}{\partial z}\right)}^{2}\right]-\frac{1}{a}\left(\frac{\partial \theta }{\partial \tau }-\frac{\partial T}{\partial \tau }\right)T\right\}dxdydz$$

This formula is to obtain the volume integral with the variation principle in the domain R. When the temperature T is taken as a known temperature $T_{b}$ on the boundary, this leads the functional represented by I(T) to minimum, then the obtained temperature field satisfies the heat conduction Laplace Equation (18) in the region R, and the temperature at any point inside the specimen, that is, the unstable temperature field can be obtained.

Divide the solution area into a numerical model with finite elements [42], then the nodes of one is i,j,m,⋯,p, the node temperature $T_{i}\left(\tau \right),T_{j}\left(\tau \right),\cdots T_{p}\left(\tau \right)$ is a function of time τ, and the any point temperature in the element can be defined as:

(23) $$T_{e}\left(x,y,z,\tau \right)=\left[N\right]{\left\{T\right\}}^{e}$$

where [N] and ${\left\{T\right\}}^{e}$ are the coordinate shape function and temperature column vector of each node in the element, respectively. In the functional within the subdomain, it is assumed that the variation difference ∂T/∂τ at temperature can be ignored. In order to obtain the minimum value of the functional I(T), take the derivative of the integral. It should be written as:

(24) $$\frac{\partial I_{e}}{\partial T_{i}}=h_{ii}^{e}T_{i}+h_{ij}^{e}T_{j}+h_{im}^{e}T_{m}+\cdots +r_{ii}^{e}\frac{\partial T_{i}}{\partial \tau }+r_{ij}^{e}\frac{\partial T_{j}}{\partial \tau }+r_{im}^{e}\frac{\partial T_{m}}{\partial \tau }+\cdots -f_{i}^{e}\frac{\partial \theta }{\partial \tau }=0$$

where $h_{ij}^{e}=\underset{\Delta R}{∭}\left(\frac{\partial N_{i}}{\partial x}\frac{\partial N_{j}}{\partial x}+\frac{\partial N_{i}}{\partial y}\frac{\partial N_{j}}{\partial y}+\frac{\partial N_{i}}{\partial z}\frac{\partial N_{j}}{\partial z}\right)dxdydz$, $f_{i}^{e}=\frac{1}{a}\underset{\Delta R}{∭}N_{i}dxdydz$ and $r_{ij}^{e}=\frac{1}{a}\underset{\Delta R}{∭}N_{i}N_{j}dxdydz$ are the elements of the conducting matrix.

The conducting matrix is supposed as $H_{ij}=\sum_{e}h_{ij}^{e},R_{ij}=\sum_{e}r_{ij}^{e},F_{ij}=\sum_{e}\left(-f_{ij}^{e}\right)$. With time points contiguous $\tau =\tau_{n}$ and $\tau =\tau_{n+1}$, substituting the matrixes into Equation (21) for the connection between time and temperature known as $\Delta T_{n}=T_{n+1}-T_{n}=\Delta \tau_{n}{\left\{\frac{\partial T}{\partial \tau }\right\}}_{n+1}\Rightarrow {\left\{\frac{\partial T}{\partial \tau }\right\}}_{n+1}=\frac{T_{n+1}-T_{n}}{\Delta \tau_{n}}$, the temperature field leads into the equation as follows:

(25) $$\sum_{e}\frac{\partial I_{e}}{\partial T_{i}}=\left[H\right]\left\{T_{n+1}\right\}+\left[R\right]\frac{T_{n+1}-T_{n}}{\Delta \tau_{n}}+\left\{F_{n+1}\right\}=0\Rightarrow \left(\left[H\right]+\frac{1}{\Delta \tau_{n}}\left[R\right]\right)T_{n+1}-\frac{1}{\Delta \tau_{n}}\left[R\right]T_{n}+\left\{F_{n+1}\right\}=0$$

With equations above, the only unknown quantity is the temperature $T_{n+1}$ on each point in the element, so for the temperature field $\left\{T_{n+1}\right\}$ of the element node at $\tau =\tau_{n+1}$, it is that to solve the linear system of equations with respect to $T_{n+1}$. The formation of ice crystals is controlled by the temperature field and pore structure, and then affected by pore water migration and stress distribution. This is the basic idea to obtain the temperature field of mortar.

## 5. Numerical Simulation for Saturated Mortar

Equations (9), (17) and (18) derived above are very complicated, and it is almost impossible to obtain theoretical solutions after simultaneous operation. Therefore, numerical solutions are sought by means of finite element programs based on partial differential equations to predict the temperature distribution, strain and frozen water content in specimens. In this paper, the latent heat of phase transition is incorporated into the heat conduction equation in the process of pore water freezing to simulate the coupling calculation with seepage, temperature and stress.

Geometric model

**Figure 2:**
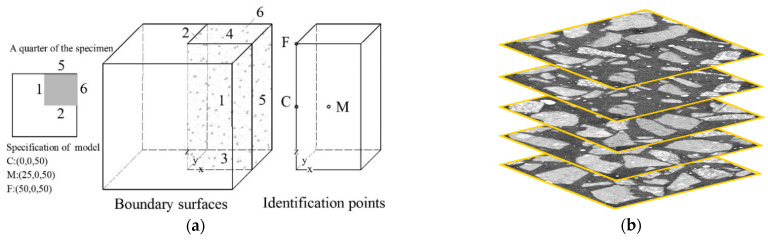
(**a**) The schematic diagram of specimen; (**b**) The two-dimensional scanning image.

**Figure 3:**
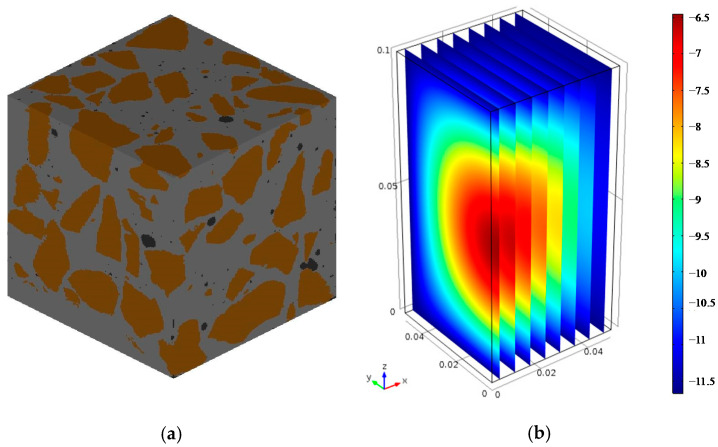
(**a**) The reconstructed three-dimensional structure; (**b**) The temperature field distribution.

The model specimen adopted was used to simulate the standardized laboratory rapid freezing-thawing test, which is immersed in water for seven days to achieve saturation before rapid freeze-thaw cycles at the age of 28 days. A 50 mm × 50 mm × 50 mm cube of concrete was cut from a 100 mm × 100 mm × 100 mm with a water-cement ratio of 0.6. Based on the CT image scanning technology of the concrete specimen, 1000 slice images of 50 mm × 50 mm were formed. The image signal, which consists of cement paste, hydration products and cement particles was discretized separately and preprocessed by MATLAB. After that, the two-dimensional image was reconstructed into a three-dimensional structure numerical model to calculate the temperature distribution of the specimen in the freezing-thawing process. The two-dimensional scanning image and the reconstructed three-dimensional structure are respectively shown in Figure 2b and Figure 3. Due to the complexity of material composition, the three-dimensional structure was partitioned into one million hexahedral mesh elements in order to make the solver converge and produce solutions with high precision. The time step size was self-adaptive below 1s with the relative tolerance for convergence 0.01.

In the finite element calculation process, the initial temperature of the model was set as 10 °C in a free state without pore water pressure, and the latent heat of water-ice phase transition in the pore structure was applied to the calculation area as a distributed heat source. Furthermore, 1 and 2 are symmetry surfaces of the original specimen, 3~6 are boundary surfaces communicated with external, the temperature of which decreased gradually from initial temperature $T_{a}=10$ °C to the lowest temperature −20 °C within 3 h except the top face, which could be expressed by $T_{b}=T_{a}-t/360$ with the cooling rate 10 °C/h. The schematic diagram and two-dimensional scanning image are shown in Figure 2a with the center point C, surface point F and the intermediate point M. The hysteresis decreases with the distance from the center point C.

Input Data and Boundary conditions

The relevant parameters for temperature field analysis are shown in Table 1. The thermal conductivity is extracted from the literature published by Zuber’s study on the volume instability upon freezing [19], the density, total porosity, and thermal conductivity, and heat capacity for water/ice are from the research on ice physics by Bazant [17], and the latent heat of water is adopted from G. Fagerlund [24]. The numerical analysis for the motor not only can be used for the size influence on temperature hysteresis effect intuitively, but also a method to research for the temperature stress sensitivity effected by the physical parameters, such as elastic modulus, Poisson’s ratio, thermal conductivity and specific heat, and linear expansion coefficient. The results of the macro numerical analysis show the importance of rationality input parameters on the frozen damage process. The boundary conditions and pore water pressure are indicated in Table 2.

The temperature field calculation results of the specimen are shown in Figure 3.

### 5.1. Temperature and Freezing Rate

**Figure 4:**
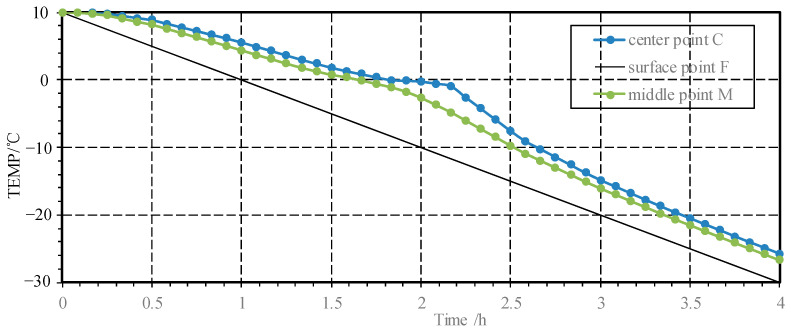
Temperature distribution of Mortar specimen.

The numerical model was established with the material parameters and boundary conditions, and the temperature changes at the center point C, the half-center point M and the surface point F of the model were derived through the finite element method. As can be seen from the process diagram Figure 4, the temperature gradually decreased from inside to outside at the same time, and compared with the surface of the specimen, the temperature drop at the center point C lagged significantly.

The difference in the initial stage is mainly caused by the initial temperature, subsequently it is mainly affected by the size and the thermal conductivity coefficient of mortar, which makes the difference between them gradually increase. After the temperature drops to 0 °C, the temperature curve appears to stay in a long platform due to the released heat from freezing. The temperature difference of internal and external reaches the maximum to 10.73 °C as the boundary temperature drops to −10 °C, indicating that latent heat is released at this stage, which remains the temperature in local areas at a high level during the freezing transformation. Along with the completed freezing, due to the large temperature difference and thermal conductivity, the temperature curve will decrease rapidly until it is consistent with the external cooling rate. The stable material parameters and boundary conditions keep the temperature difference at about 8°C between center and surface.

**Figure 5:**
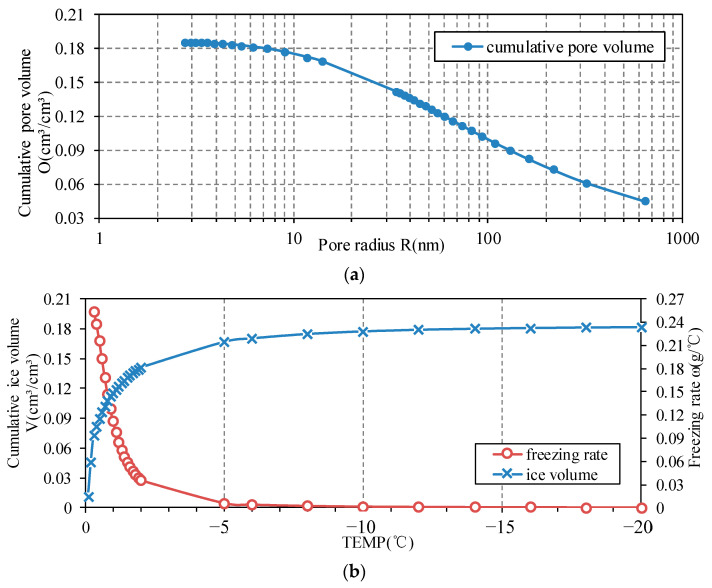
(**a**) Temperature distribution of Mortar specimen; (**b**) Freezing volume and freezing rate.

According to the third section, the porosity of the mortar specimen is 0.1861. As the overall temperature decreases, the critical pore radius of phase transition gradually decreases, which indicates that the ice crystal is framed based on the pore structure and forms in the large pores until the tip of the ice body reaches the equilibrium of the curved liquid surface. As a result, the size of the equilibrium ice crystal continues to decrease, it grows into the smaller pores and increases the overall freezing amount. The freezing volume in the specimen is therefore affected by both boundary temperature and pore structure, which is the difference between the accumulated pore volume over the critical radius and the accumulated volume of attached water. The pore structure and freezing rate are shown in Figure 5. According to the calculated data, when the temperature is −5 °C, the cumulative pore volume of about 15 nm is 0.168, accounting for more than 85% of the total pores in the model, 15 nm is close to the lower limit of the pore radius identification capability of MIP technology.

### 5.2. The Coupling Strain from Freezing

The thermodynamic equilibrium state is always effective for capillary and closed pores before and after freezing. Although the crystallization of pore water will produce 9% expansion and cause unfrozen water flow, frost heave deformation generated is small enough to be negligible with medium saturation upon most occasions, and most of them would be reversible in the process of heating and melting, at which time, the specimen tends to shrink without expansion. In this study, it was assumed that the specimen was in a saturation state to ensure that all pores were filled with water, so that the deformation occurred during the phase transition exceeded the tensile strength of the material and plastic failure occurred.

Beginning at 10 °C, it is known to all that the temperature was lowered stepwise with a defined cooling amplitude and rate, pore water does not freeze above 0 °C, and the specimen will be in a continuous contraction state resulting in negative pore pressure, which is mainly affected by the linear expansion coefficient of the system, otherwise the temperature drops over 0 °C. The rapidly increasing pressure from the ice formation and the hydrostatic pressure caused by freezing progressively, make it transition to the main source of pressure leading to an obvious expansion. The maximum vertical strain $\varepsilon_{z}$ was 4.27 × 10^−6^ at approximately −10 °C.

**Figure 6:**
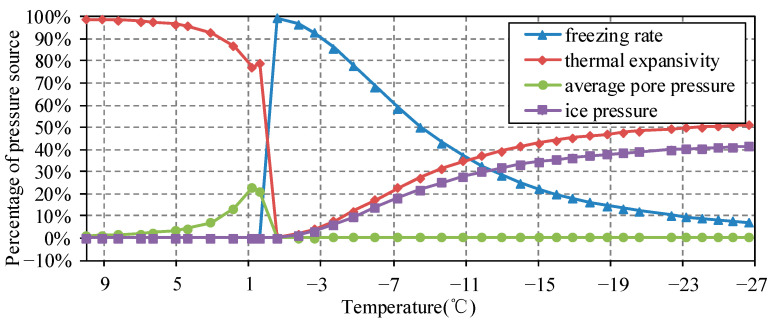
The source analysis of water pressure in low temperature process.

As the temperature continues to drop, the freezing rate slows down with the unfrozen water in the pores decreasing, and the pressure gradually releases as discussed in the previous section. The tension of the water-ice and linear expansion coefficient of the system gradually increase. Next, pure thermal contraction occurred as the third part of the pressure dominated. The contribution of water pressure source to the average pore water pressure is shown in Figure 6.

**Figure 7:**
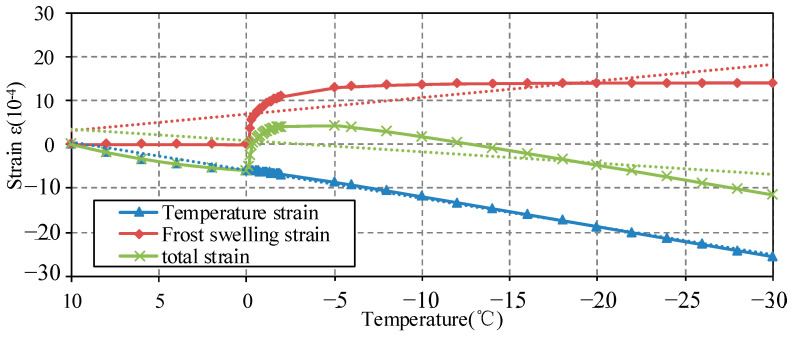
The low-temperature strain process of mortar specimen.

The temperature distribution was obtained from the control equation in Section 3, from which the freezing rate and ice volume in pores can be deduced. Among the sources of water pressure, the freezing rate and linear expansion coefficient of the system consist of the main factors affecting the strain. The accumulated temperature strain and accumulated frost heaving strain at the surface point F are shown in Figure 7.

As seen from Figure 7 in the positive temperature zone from 10 °C to 0 °C, the total strain of surface point F reduces with the decrease of the external temperature. The strain is distinctly affected by the system linear expansion consisting of mortar and pore water. As the linear expansion coefficient of the specimen changes with the water expansion coefficient, the pore pressure is in the state of negative pressure. When the temperature drops to 0 °C, the pore water freezes and expands, resulting in pore pressure acting on the mortar specimen to counteract the cold shrinkage deformation, so that the expansion amount of the specimen increases rapidly. At the same time, due to the expansion coefficient of ice 159 × 10^−6^°C^−1^, which is much higher than the expansion coefficient of mortar material 30 × 10^−6^°C^−1^ (−10 °C), the freezing amount continues to increase in the new cold shrinkage space. With the freezing completed in large pores, the freezing rate slows down at the same cooling rate. When the temperature drops to −10 °C, the maximum strain at point F reaches 4.27 × 10^−4^. After that, the total strain is again controlled by the linear expansion coefficient, and the strain decreases gradually with the temperature.

## 6. Further Discussion

Concrete is a quasi-brittle material, and its freezing is a coupling process involving stress, seepage and temperature field. Due to the heterogeneous nature of components, the pores with different sizes, shapes and connectivity will cause tensile stress during the frost process, which leads to the further expansion and extension of micro-cracks virtually. The crack width, density and connectivity of the surrounding concrete will increase with the increased number of freeze-thaw cycles [43]. However, it is simplified to simulate a single frost of a closed spherical hole without rupture damage in this paper. In the partially saturated pore system without ice formation, the relative water pressure is always less than or equal to zero, so the initial condition of pore water pressure can be assumed to be approximately 0 MPa. The resulting negative pressure compresses the porous material, leading to a slight overestimation of the mortar expansion.

Based on the poromechanics considering the effect of phase change latent heat, the calculation shows that the results obtained from the developed mortar specimen multi-field coupling control equation can be used as a reference to the frost concrete which conforms to the basic physical law.

For conventional concrete, the tensile strength is generally about 1/10 of the compressive strength, and the long-term tensile limit deformation is about 1.2~2.0 × 10^−4^. Without considering the difference influence of temperature between laboratory and field, the mortar specimens will face the risk of failure in this paper [44].

It can be known from the temperature calculation, the frost depth increases with the temperature difference directly, thus, the specimen is affected by freezing at a depth of 0.1 m in the simulation. The temperature stress caused by the temperature difference of 20 ℃ is enough to destroy the concrete specimen calculated on the basis of elastic-plastic. In order to control the damage degree subjected with the changeless environmental temperature and building size, several aspects could be taken into consideration.

- Control the size of some high-performance concrete structures within the frost depth to avoid the temperature difference large enough to cause freezing damage.
- The main factor affecting frost resistance of hardening cement is the water-cement ratio. The strength or toughness can be enhanced by reducing water cement ratio appropriately.
- Update the concrete mix ratio as needed and adopt various air-entraining admixtures to improve the thermodynamic performance.
- Sticking the insulation board to reduce the direct influence of large temperature difference on concrete for antifreeze and heat preservation.

## 7. Conclusions

The bulk instability of concrete is caused by mass transfer between water and ice at temperatures below freezing point. Although the governing equations of the freezing process are known, a comprehensive fully coupled THM model is not yet available. In this paper, based on the principle of equilibrium, continuity and energy, the THM coupling model of concrete multiphase porous media is established considering the interaction between the change of water pressure and ice transformation with the latent heat in concrete.

In order to verify the proposed THM coupling model, a finite element program is used to realize the numerical solution of the three-field coupled equations, and the three-dimensional finite element analysis of the freezing process is carried out for the variation trend and peak value of the frozen strain. The numerical model can be used to evaluate the effects of pore size distribution, cooling rate, permeability, cross-section size and shape on freezing process.

The calculation method proposed in this paper laid a foundation for developing comprehensive tools for numerical simulation of frost damage deterioration in concrete. Because of the differences between the site environment and standard freeze-thaw test, freeze-thaw test data are difficult to apply to the durability evaluation. The future research, on the one hand, should pay attention to the relationship among the numerical simulation, laboratory test and field, on the other hand, the research method should be extended to the micro-scale numerical simulation to obtain the effective feedback of the real pore structure subjected to the subfreezing process, so as to form a theoretical and practical system that can be interpreted from the micro level to the macro level.

Finally, in order to predict the large residual deformation caused by material nonlinearity, the nonlinear constitutive relationship and damage behavior law of concrete should be considered in the future. Simulating crack propagation during concrete freezing can be a challenging task and is beyond the scope of this study. Considering the above phenomena, it is worthwhile to continue to develop the model.

## Figures and Tables

**Table 1:** Input material parameter adopted for computation.

Properties of Water	Value	Properties of Mortars	Value
Water density $\rho_{l}$ [kg/m^3^]	1000	Density $\rho_{m}$ [kg/m^3^]	2140
Water specific heat $C_{l}$ [kJ/(kg·K)]	4.22	Elastic modulus E (GPa)	10.1
Water conductivity $\lambda_{l}$ [W/(m·K)]	0.55	conductivity $\lambda_{m}$ [W/(m·K)]	0.93
Water compressibility $K_{w}$ (GPa)	2	specific heat $C_{m}$ [kJ/(kg·K)]	0.84
Ice density $\rho_{s}$ [kg/m^3^]	0.916	Biot’s coefficient $b_{i}$ [-]	0.461
Ice specific heat $C_{s}$ [kJ/(kg·K)]	2.11	Permeability D [m^2^]	8.343 × 10^−21^
Ice conductivity $\lambda_{s}$ [W/(m·K)]	2.2	Latent heat $H_{l}$ [kJ/kg]	333.5
Ice compressibility $K_{i}$ (GPa)	8	Total porosity n	0.186

**Table 2:** Boundary conditions of temperature field analysis.

Item	Symbol	Boundary Conditions
Initial TEMP	$\;T_{a}$	$T_{a}=10$ °C
Cooling fall rate	ω	10 °C/h
TEMP drop	A	10→−20 °C, A=30 °C, lasted for 3 h
Cool boundary	face	Symmetric faces 1 and 2, Heat flux = 0
		Exposed surface 3, 4, 5, 6. $T=T_{b}$
Fixture boundary	face	Symmetric faces 1 and 2, Bottom face 3
Heat sources (latent heat)	H	Distributed in the calculation domain $H=H_{l}\times {\dot{\omega }}_{l\to s}$

## Data Availability

The data presented in this study are available on request from the corresponding author. The data are not publicly available due to that they involve confidential information of the project.
